# Mapping and characterization of G-quadruplexes in *Mycobacterium tuberculosis* gene promoter regions

**DOI:** 10.1038/s41598-017-05867-z

**Published:** 2017-07-18

**Authors:** Rosalba Perrone, Enrico Lavezzo, Erika Riello, Riccardo Manganelli, Giorgio Palù, Stefano Toppo, Roberta Provvedi, Sara N. Richter

**Affiliations:** 1 Department of Molecular Medicine, University of Padua, via Gabelli 63, 35121 Padua, Italy; 2 Department of Biology, University of Padua, via Ugo Bassi 58/b, 35121 Padua, Italy

## Abstract

*Mycobacterium tuberculosis* is the causative agent of tuberculosis (TB), one of the top 10 causes of death worldwide in 2015. The recent emergence of strains resistant to all current drugs urges the development of compounds with new mechanisms of action. G-quadruplexes are nucleic acids secondary structures that may form in G-rich regions to epigenetically regulate cellular functions. Here we implemented a computational tool to scan the presence of putative G-quadruplex forming sequences in the genome of *Mycobacterium tuberculosis* and analyse their association to transcription start sites. We found that the most stable G-quadruplexes were in the promoter region of genes belonging to definite functional categories. Actual G-quadruplex folding of four selected sequences was assessed by biophysical and biomolecular techniques: all molecules formed stable G-quadruplexes, which were further stabilized by two G-quadruplex ligands. These compounds inhibited *Mycobacterium tuberculosis* growth with minimal inhibitory concentrations in the low micromolar range. These data support formation of *Mycobacterium tuberculosis* G-quadruplexes *in vivo* and their potential regulation of gene transcription, and prompt the use of G4 ligands to develop original antitubercular agents.

## Introduction

*Mycobacterium tuberculosis* (*Mtb*) is the causative agent of tuberculosis (TB), a disease existing for millennia and still remaining a major global health problem. Primary infection occurs by inhaling aerosol particles containing bacteria. *Mtb* is able to replicate inside alveolar macrophages and inflammatory cells recruited at the infection site determine formation of a histological pulmonary lesion named granuloma. In most cases *Mtb* is never cleared and survives inside granulomas in a non-replicative and non-infectious state known as latency^1^. Around one third of the world’s population is affected by latent TB. Latently infected individuals have 5–15% probability to develop the active disease during their lifetime. According to the 2016 World Health Organization (WHO) report, 10.4 million new TB cases were estimated worldwide, with 480,000 new cases of multidrug-resistant TB (MDR-TB) and 1.4 million deaths. In addition, the emergence of extensively drug–resistant TB (XDR-TB) and totally drug–resistant TB (TDR-TB) is becoming one of the biggest threats to public health and TB control programs^2^. Therefore, new insights into *Mtb* physiology are required to better characterize the pathogenesis mechanisms that *Mtb* exploits to survive and persist in its host, in order to individuate strategies to eradicate this ancient pathogen.

G-quadruplexes (G4s) are nucleic acids secondary structures that may form in single-stranded G-rich sequences under physiological conditions^3^. Four Gs bind via Hoogsteen-type hydrogen bonds base-pairing to yield G-quartets, which stack to form the G4. The presence of K^+^ cations specifically supports G4 formation and stability^4^. Based on the strand orientation, G4s can adopt three main topologies: parallel, antiparallel, and hybrid-type structures. Stability studies about the formation of G4s have demonstrated that these non-canonical DNA secondary structures are able to destabilize the double helix, since many G4 structures are thermodynamically more stable than double stranded DNA and their unfolding kinetics are significantly slower^5, 6^. In eukaryotes G4s have been reported to be involved in key regulatory roles, including transcriptional regulation of gene promoters and enhancers, translation, chromatin epigenetic regulation, DNA recombination^6–10^. Expansion of G4-forming motifs has been associated with relevant human neurological disorders^8, 11^. Formation of G4s *in vivo* has been consolidated by the discovery of cellular proteins that specifically recognize G4s^12, 13^ and the development of G4 specific antibodies^14, 15^. In viruses G4s have been implicated in key steps^16^: in the human immunodeficiency virus, the presence of functionally significant G4s^10, 13, 17–19^ and their targeting by G4 ligands with consequent antiviral effects^10, 20, 21^ have been reported. G4s have been also discovered in herpesviruses^22–25^, SARS coronavirus^26^ and human papilloma, Zika, Ebola and hepatitis C virus genomes^27–30^.

In prokaryotes, G4 sequences have been reported in *Escherichia coli* ^7, 31, 32^, *Deinococcus radiodurans* ^33–35^ *Xanthomonas* and *Nostoc sp* ^36^. Evidence of bacterial enzymes that process G4s, such as Pif1 and RecQ helicases, has been provided in *Escherichia coli*, *Clostridium difficile* and *Bacteroides sp* ^37–42^. Bacterial G4s have been implicated in antigenic variation of the cell-surface pilin proteins of *Neisseria gonorrhoeae* ^43–46^. In *Mtb*, whose genome is 65% GC rich, previous bioinformatics analysis identified more than 10,000 motifs with the potential to fold into G4 structures^32^. Additionally, evidence for the presence of a specific helicase that targets G4s (DinG) and for a G4 aptamer that inhibits a polyphosphate kinase involved in the inorganic polyphosphate intracellular metabolism has been provided in *Mtb* ^47, 48^.

The involvement of G4 structures in several human diseases propelled the development of small molecules directed against G4s^9^. Aromatic cores with protonable side chains, such as the acridine, BRACO-19^49, 50^ and water-soluble naphthalene diimides (NDIs)^21, 51–56^, specifically bind the G4 conformation. So far, the vast majority of molecules has been tested against cellular G4s implicated in tumor pathogenesis: some compounds showed interesting antiproliferative properties^57^; in particular, quarfloxin proceeded into phase II clinical trials, but its limited bioavailability prevented further progress^58^. In bacteria, N-methyl mesoporphyrin has been shown to attenuate *Deinococcus* resistance to radiation^33^; to our knowledge no other G4 ligand has been so far tested in bacteria.

To search for G4 motifs in *Mtb*, we have implemented a tool able to scan the whole genome and rank potentially interesting G4s according to their score. Only high scoring hits close to known transcription start sites (TSS) were considered. Four G4 sequences, close to the TSS of genes with known function, were selected and their G4 folding confirmed in solution. Two G4 ligands stabilized the selected G4s and inhibited bacterial cells growth with minimal inhibitory concentrations (MIC) in the low micromolar range.

## Results and Discussion

### Identification of putative G4 motifs in the promoter region of *Mtb* genes

To detect the presence of putative G4 motifs, the *Mtb* genome was scrutinized *in silico* assessing various lengths of G-islands and loops (Supplementary Figures 1a and b). A G4 was reported when at least four consecutive G-islands (*n* = 4) were identified. We also defined two parameters, *l* and *d*, corresponding to the minimal length of a G4 homopolymeric G-island and the maximum allowed distance between consecutive G-islands, respectively. Different combinations of *l* and *d* parameters were applied to allow the detection of G4 motifs with increasing stringency (*i.e*. 2 ≤ *l* ≤ 5 and *d* = 7, 11, and 15); we chose G4s with loop length up to 15 nucleotides since it has been reported that they can fold into stable G4s^59^. Computational searches have detected a high concentration of G4 motifs near promoter regions both in eukaryotic and prokaryotic genomes and in some cases a possible role of G4 motifs in transcription regulation has been reported^60^. For this reason and because of the abundance of GC content in *Mtb*, we restricted G4 analysis to regions close to transcription start sites (TSS). A short and a long score were computed considering 15 and 50 nucleotides, respectively, both upstream and downstream of the G4 motif, according to Beaudoin *et al*.^61^ (Table 1).

**Table 1:** Number of putative G4s in both strands of the *Mtb* genome within 50 nts upstream of a primary TSS.

	Pattern (l_n_d)	Total G4s	G4s in TSS
Forward strand	2_4_15	33081	1115
	2_4_11	30561	805
	2_4_7	21186	426
	3_4_15	902	29
	3_4_11	495	13
	3_4_7	223	4
	4_4_15	82	0
	4_4_11	10	0
	4_4_7	5	0
	5_4_15	0	0
	5_4_11	0	0
	5_4_7	0	0
Reverse strand	2_4_15	33061	1122
	2_4_11	31109	845
	2_4_7	21747	479
	3_4_15	1074	33
	3_4_11	574	14
	3_4_7	252	6
	4_4_15	21	1
	4_4_11	4	0
	4_4_7	1	0
	5_4_15	1	0
	5_4_11	0	0
	5_4_7	0	0

Position of the found G4s in the *Mtb* genome is available in Supplementary Files S1a and S1b.

The genomic coordinates of the predicted G4s both in the forward (Supplementary File S1a) and in the reverse strand (Supplementary File S1b) were intersected with the putative gene promoters, inferred by considering 50 nt upstream of the known primary TSS^62^ (Table 1 and Supplementary File S2 “Primary TSS”). The G4 motifs overlapping promoter regions were ranked by the short and long scores (Supplementary File S2 “G4 overlapping promoters”). As expected, the amount of detected G4 motifs decreased with the stringency of the searching parameters (i.e. longer G-islands and shorter distance between them). Moreover, the distribution of the predicted G4s was homogeneous in the two strands of the genome, with a slight prevalence of the reverse strand in six categories (out of 12) as opposed to four categories, which were more abundant in the forward strand (Table 1). To note that both the forward and reverse strand, depending on the gene, can be the coding strand in transcription.

### Genes with putative DNA G4 forming sequences in *Mtb*

Based on the described bioinformatics analysis, we identified 45 genes with a putative G4, upstream or overlapping their TSS, with at least 3 Gs in each island (therefore with the ability to form at least a three-stacked G4) and a short or long score ≥ 2 (Table 2 and Supplementary File S2 “Candidate genes”). This threshold was chosen according to Beaudoin *et al*.^61^, which did not validate G4s with lower score. These genes were classified according to their functional category as reported in TubercuList^63^. In addition, a *de novo* function prediction based on Gene Ontology (GO) annotations was performed with the online server Argot2.5^64^ to expand already available annotations and potentially define functions for those genes that are still hypothetical/unknown (Supplementary File S2 ‘Function prediction’). Globally, 35 genes out of 45 were annotated with at least one GO term: 8 of them had been previously unannotated, while the others were confirmed or expanded (Supplementary File S2 “Candidate genes”). We found that most G4s were distributed among the following functional categories: “cell wall and cell processes”, “intermediary metabolism and respiration”, “regulatory proteins”, and “conserved hypotheticals” (i.e. conserved proteins with no confirmed known function).

**Table 2:** G4 sequences upstream or overlapping TSS in the *Mtb* genome, forming G4s with at least three stacked tetrads (at least 3 Gs in each G-rich island) and with short or long score ≥ 2.

Rv number	Gene name	Nts to TSS^a^	G4 sequence
Rv0011c	crgA	−14	**GGG**CA**GGG**TGTT**GGG**T**GGG**
Rv3779		−35	**GGG**AAGCCC**GGG**C**GGG**CTGGG*
Rv0284	eccC3	−18	**GGG**CGCC**GGG**TCGTTGTTCT**GGG**TGTCGGATACC**GGGG**
Rv3208		−35	**GGG**ATAGTTTGTT**GGG**TGTTGCATTC**GGG**CGCGCCA**GGG**TCGCGACCCGGG*
Rv2639c		−26	**GGG**TGAC**GGG**AAGCATTT**GGGG**TGCGCGATTGGTT**GGGGGG**CGGCA**GGG**
Rv0713		−47	**GGGGGG**CTTGGCTTTTT**GGGG**CAACCGGACCAGCGAGGG*
Rv1338	murI	1	**GGG**CTTTTTGTGCGCAA**GGG**AT**GGG**ATATCGTCATT**GGG**
Rv3435c		35	**GGG**CCGGAACGCACAAGT**GGG**C**GGG**TAGCGAGTT**GGG**
Rv2597		−25	**GGG**ACC**GGGGG**TCACAAC**GGG**CGAGTTGTCCGGCCGGG*
Rv3802c		18	**GGG**CGAAGCCGCGTAGC**GGG**CCGGTACCGTAGA**GGG**AGT*GC* *GG*CAAC**GGG**C**GGG**
Rv2732c		−22	**GGG**CAGCC**GGGG**CGCGCCGTCGGCCT**GGG**CATGCCT**GGGG**TC**GGG**
Rv1539	lspA	−12	**GGGG**TCT**GGG**C**GGG**CCATATCGGCCCTA**GGGG**
Rv3484		−24	**GGG**C**GGG**TACC**GGG**A**GGG**TTAGCGGG*
Rv0150c		−12	**GGG**TTT**GGGG**TTCACCGCGAT**GGG**TGAGTAT**GGG**
Rv2030c		−17	**GGGG**AAGA**GGG**ACCGC**GGG**T*GG* *CG*CTGAAC**GGG**AAGGG*
Rv2405		−48	**GGGGG**TTGAC**GGG**TATCCA**GGG**TATCCGCGTC**GGG**
Rv3404c		44	**GGG**TGAGCT*GG* *TG*TT**GGGGG**CTCCGCTGAT**GGG**CGCT**GGG**CAGGCT*GG* *CG* **GGGG**
Rv2559c		30	**GGGGG**T**GGG**CCGTAGCCT*GG* *T* *GG* *CG* **GGGG**A*GG* *CG*CTCCGTAGCC**GGG**CGGC**GGG**
Rv3207c		34	**GGG**ATAGTTTGTT**GGG**TGTTGCATTC**GGG**CGCGCCA**GGG**TCGCGACCC**GGG**
Rv2308		−29	**GGG**ACGC**GGGGG**TGGCCCCGCTCTAT**GGGG**TGAGCCGGG*
Rv0750		21	**GGG**ACTAAACTATCTA**GGG**CAAGTGC**GGG**CCATAGT**GGG**
Rv0471c		20	**GGGGG**CAGGTCTAGGCTTTA**GGG**ATGCCCGACGC**GGG**CGC**GGG**
Rv0628c		−27	**GGG**CCGCGATCGCACGCC**GGG**CGGTGC**GGG**CCAGC**GGG**
Rv1253	deaD	−40	**GGGG**CA**GGG**T*GG* *TG*ACCACACACC**GGG**CACCGTACCGCCATC**GGG**CCCGC**GGG**
Rv2979c		−47	**GGG**CGACGACGCATC**GGGGGG**TGCCAGCTGTTGC**GGG**
Rv0245		−4	**GGG**TT**GGG**TA**GGG**TT**GGG**
Rv1121	zwf1^	−8	**GGG**TTGTC**GGG**CCAAT**GGG**CTA**GGG**
Rv0392c	ndhA^	−47	**GGG**CCTTGT**GGG**CCTTGT**GGG**CCTTGT**GGG**
Rv2457c	clpX^	10	**GGGGGG**CC*GG* *AG*CAAGC**GGG**TAGCGTC**GGGG**CATACAC**GGGG**
Rv1419		40	**GGGG**AAAT**GGG**TGAATTACGGTTGGT**GGG**C*GG* *TG*TGCTCC**GGG**
Rv1327c	glgE	12	**GGG**TGTGATCGGATACTA**GGG**T**GGG**TATC**GGG**
Rv0851c		18	**GGG**TGACTGCCTGAAATA**GGG**TTGCGTGCT*GT* *GG*AC**GGG**TTTCCC**GGG**
Rv3634c	galE1	−4	**GGG**CGACCC*GA* *GG*CATAC**GGGG**CGCTGGCAT**GGG**CCGCCGGTATGGT**GGG**
Rv2847c	cysG	−30	**GGG**GACC**GGG**CGCCGC**GGG**TCGCCACCATCA**GGG**
Rv0896	gltA2	−23	**GGG**ATGACCCGCCTGCCC**GGGG**T**GGGG**TCTCTGGCACCAT**GGG**
Rv2367c		−34	**GGG**TC**GGGG**CTGAATC**GGG**CGGCTCGGC**GGG**
Rv0166	fadD5	−36	**GGG**TC**GGG**CC**GGG**ATTGCC**GGGG**ACTTGCC**GGGG**GCTTGGC**GGGGG**
Rv1704c	cycA	−13	**GGG**CACCGGTAC**GGGGGG**TGC**GGG**TCCCCGCTAC**GGG**TTCC**GGG**
Rv0339c		−14	**GGGG**CGCCGTTA**GGGG**ATGGCCGCATTA**GGGG**AAATGC**GGGG**CTGC**GGG**AC**GGG**CT**GGGG**
Rv1049	mosR^	−38	**GGG**CTAGCTCTA**GGGGG**CA**GGG**CTTTGAC**GGG**
Rv0238		−7	**GGG**TTAGATAGAC**GGG**CTACA**GGGG**CCCAAAA**GGGG**
Rv1152		41	**GGG**TAC*GT* *GG* *AG*CTGC**GGG**ATTGGTTAC**GGG**TCGACGTGAAGGC**GGG**
Rv1151c		−32	**GGG**TAC*GT* *GG*AGCTGC**GGG**ATTGGTTAC**GGG**TCGACGTGAAGGC**GGG**
Rv2021c		−36	**GGG**TGATATTCCTCC**GGG**TAAGAGCAGC**GGG**CGACGGGG*
Rv1082	mca	−21	**GGG** *GGTG*T**GGG**TCATGCCT**GGG**TTCACGCCGGC**GGG**

G tracts with at least three Gs are shown in bold. GG tracts are underlined since they may aid G4 folding. Tracts with the potential to form a bulged G4 (*i.e*. GXGG, where X is any of the three remaining bases) are additionally shown in italics. The symbol^ indicates genes, the corresponding G4 sequences of which were chosen for further investigation. Rv number is the gene numeration in the considered reference strain H37Rv. ^a^Position of the last nt of the G4 motif with respect to the TSS. Asterisks indicate that the reported G4 sequence is in the reverse strand.

Among the identified putative G4s, the sequence upstream *rv0166* (*fadD5*) (Supplementary File S2 “Candidate genes”) had been previously reported by Thakur and colleagues to fold into a G4 structure^47^. The same authors reported two additional genes to display a G4 motif; these genes are not present in our analysis since they are not associated to reported TSS^62^.

### Selected G-rich sequences in the *Mtb* genome fold into G4

Among the genes with a predicted G4 in their promoter region, we selected four candidates for further experimental validations, namely Glucose-6-phosphate dehydrogenase 1 (*zwf1*), ATP-dependent Clp protease (*clpx*), Oxidation-sensing Regulator Transcription Factor (*mosR*), and membrane NADH dehydrogenase (*ndhA*) (Table 2). The choice fell on putative G4s belonging to the most stable categories (at least three ‘Gs’ in each island and loops no longer than 11 nt), prioritizing those present in multiple categories (for instance *zwf1* has a G4 that falls both in the 3_4_7 and 3_4_11 category) with at least one score > 2 and in the promoter of genes with a known function.

**Figure 1:**
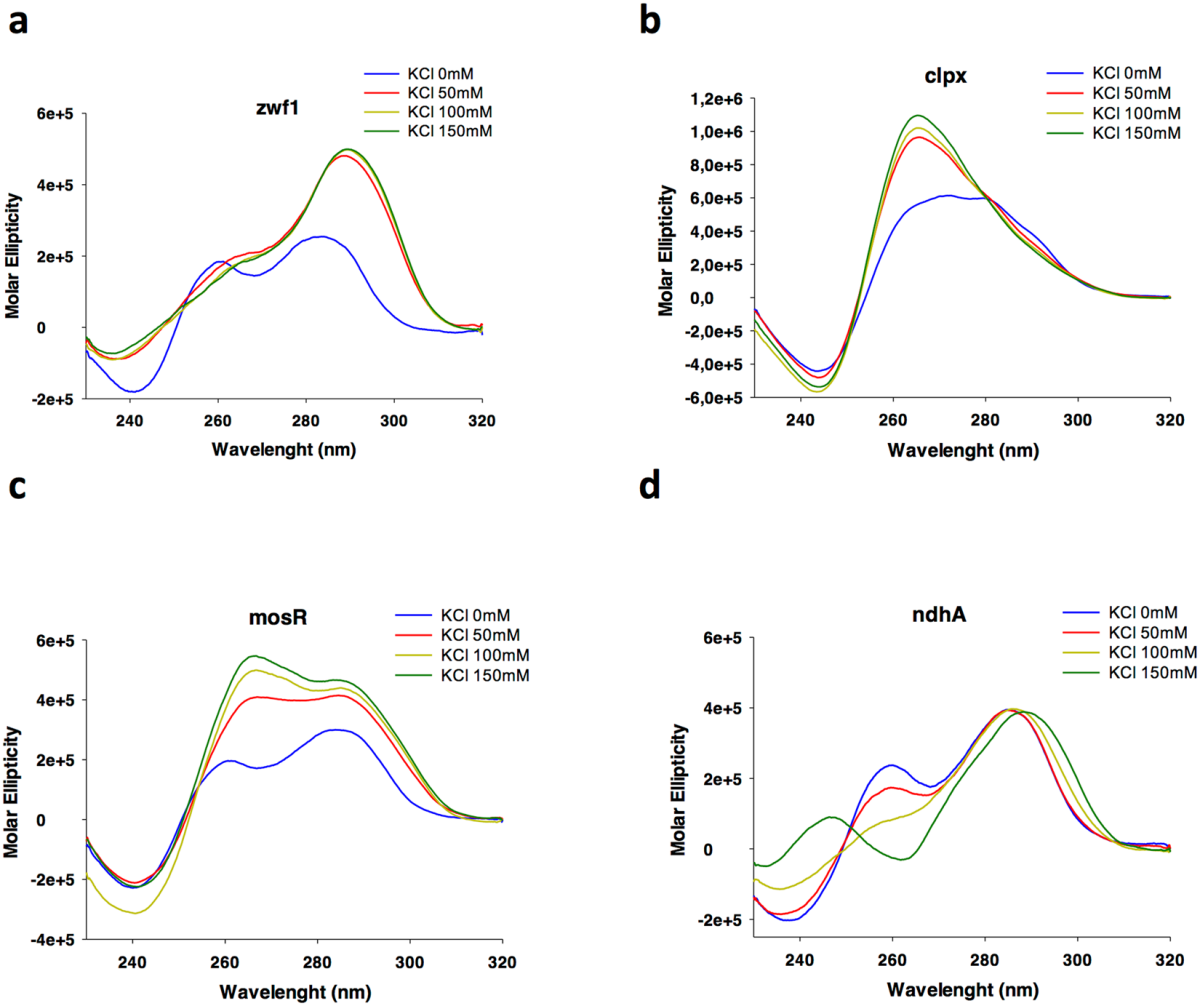
CD spectra of the putative G4 molecules of *zwf1* (**a**), *clpx* (**b**), *mosR* (**c**) and *ndhA* (**d**) in the presence of increasing KCl concentrations (0–150 mM).

G4 folding and topology was initially assessed by circular dichroism (CD) spectroscopy in the absence or presence of increasing concentrations of K^+^, since this monovalent cation is reported to stabilize the G4 conformation. All the selected molecules in the presence of K^+^ displayed the G4 CD signature (Fig. 1a–d).

The *zwf1* G4 structure exhibited a mixed-type conformation in K^+^, with a shoulder at 265 nm, a positive and a negative peak at 290 nm and 240 nm, respectively (Fig. 1a). *clpx* G4 adopted a parallel-like conformation in K^+^, with a maximum at 265 nm and a minimum at 240 nm (Fig. 1b). *mosR* G4 folded in a mixed type conformation in K^+^ showing a spectrum with two positive peaks (267 and 290 nm) and a negative peak at 240 nm (Fig. 1c). Molar ellipticity values of all these structures increased in a K^+^-dependent manner, further supporting G4 formation (Fig. 1a–c). z*wf1* and *mosR* displayed a G4-like CD spectrum (mixed-type conformation) also in the absence of K^+^, indicating high propensity to fold and stability. The *ndhA* G4 sequence transitioned from mixed-type in the absence of K^+^ to fully antiparallel (CD spectrum with two maxima at 240 and 290 nm and a minimum at 265 nm) in the presence of K^+^ 150 mM (Fig. 1d). Overall our data indicate that the selected sequences of *Mtb* can effectively fold into G4 conformations.

Stability of *zwf1*, *clpx*, *mosR* and *ndhA* G4s in the absence and presence of increasing K^+^ concentrations (50–150 mM) was assessed by melting experiments monitored by CD, calculating the melting temperatures (T_m_) according to the van’t Hoff equation (Table 3).

**Table 3:** Melting temperatures (T_m_) of *Mtb* G4 oligonucleotides (4 µM) in the absence and presence of increasing KCl concentrations (50–150 mM) and G4 ligands (16 µM).

G4	K^+^ (mM)	G-4 ligand added	T_m_ (°C)	ΔT_m_ (°C) (T_m_K^+^[50] or [100] or [150]-T_m_K^+^[0])	T_m_ (°C)	ΔT_m_ (°C) (T_m_K^+^[100]G4 ligand-T_m_K^+^[100])
zwf1	0		36.8 ± 1.27			
	50		44.2 ± 1.4	7.4		
	100		48.5 ± 0.5	11.7		
	150		52.2 ± 1.9	15.4		
	100	B19			>90.0	>41.5
	100	NDI			>90.0	>41.5
clpx	0		40.1 ± 0.1			
	50		52.0 ± 0.8	11.9		
	100		59.2 ± 0.8	19.1		
	150		74.2 ± 0.6	34.1		
	100	B19			79.9 ± 0.4	20.7
	100	NDI			79.3 ± 1.4	20.1
mosR	0		37.8 ± 0.9			
	50		49.4 ± 0.5	11.6		
	100		50.8 ± 1.7	13.0		
	150		51.7 ± 1.3	13.9		
	100	B19			(I) 68.2 ± 1.1	17.4
					(II) 50.7 ± 0.6/79.7 ± 0.7	−/28.9
	100	NDI			(I) 79.4 ± 1.0	28.6
					(II) 53.9 ± 1.6/82.5 ± 2.3	3.1/31.7
ndhA	0		60.5 ± 0.3			
	50		45.4 ± 1.6/ 71.2 ± 2.1	−/10.7		
	100		38.1 ± 0.4 / 74.4 ± 1.3	−/13.9		
	150		52.6 ± 3.1 /80.2 ± 1.3	−/19.7		
	100	B19			(I) > 90	>51.9/ > 15.6
					(II) 63.8 ± 1.5/85.2 ± 0.9	25.7/10.8
	100	NDI			>90.0	>51.9/ > 15.6

When more than one G4 species were observed in the CD spectrum (i.e. I, II), T_m_ values for each species were reported. B19 and NDI stand for the G4 ligands BRACO-19 and c-exNDI 2, respectively.

In all cases the CD signal decreased over temperature. For *zwf1*, *clpx* and *mosR* G4s a single transition between 20 °C and 90 °C was appreciable, leading to discrete T_m_ values. *ndhA* G4 showed a peculiar behaviour, with a relatively high T_m_ (60.5 ± 0.3 °C) in the absence of K^+^ and two different T_m_ values in the presence of K^+^ ascribable to two transitions due to the presence of spectroscopically distinct species in solution. Overall we observed increase of T_m_ values in a K^+^-dependent manner, indicating that G4s were stabilized by K^+^ with increase of T_m_ up to 34.1 °C (Table 3).

### Effect of G4 ligands on *Mtb* G4s

**Figure 2:**
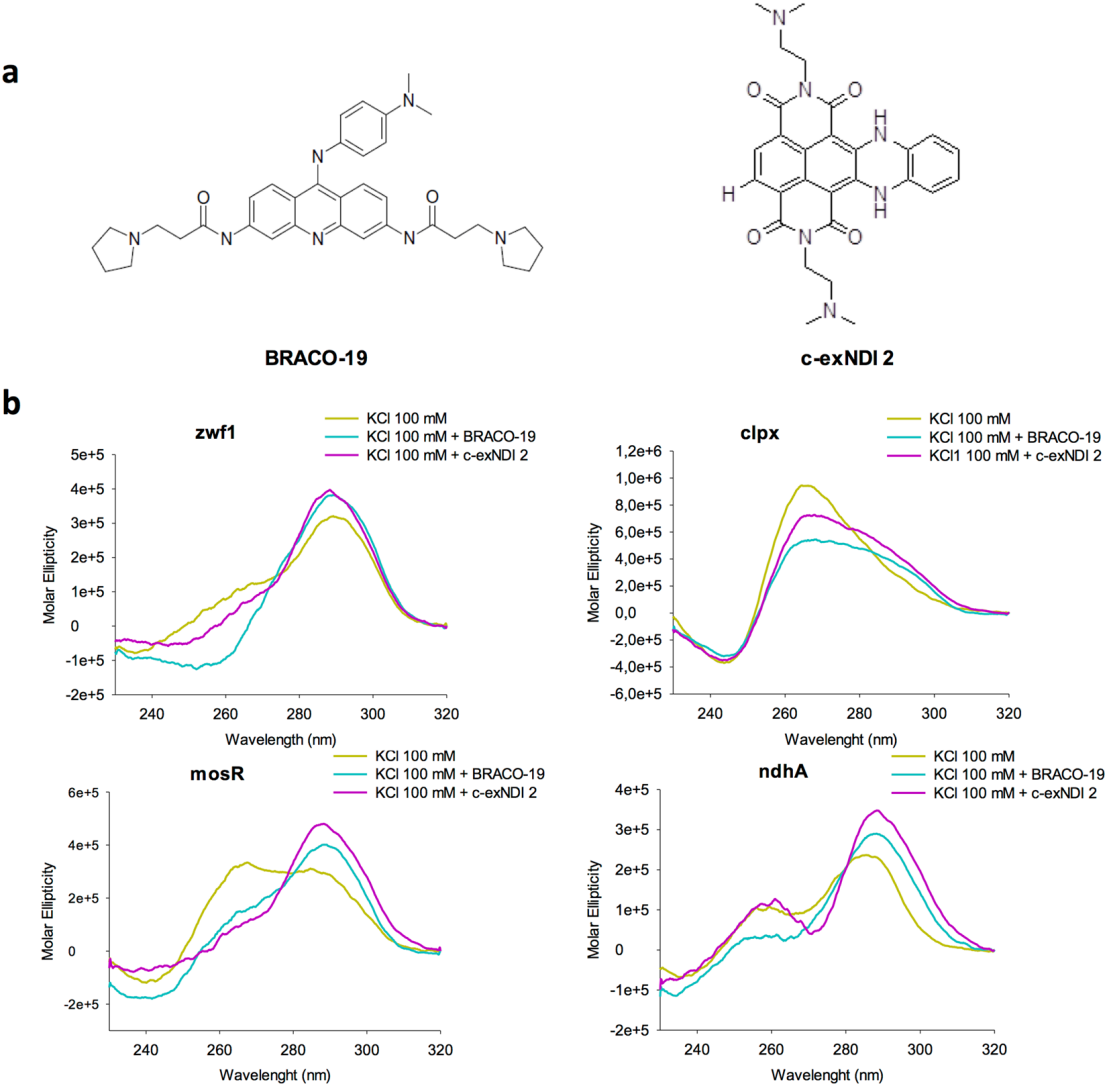
Effect of the G4 ligands BRACO-19 and c-exNDI 2 on the conformation of the selected *Mtb* G4s. (**a**) Chemical structures of the G4 ligands BRACO-19 and c-exNDI 2. (**b**) CD spectra of G4 oligonucleotides *zwf1*, *clpx*, *mosR* and *ndhA* (final concertation 4 μM) in the presence of KCl (100 mM) and BRACO-19 or c-exNDI 2 (final concentration 16 μM) to assess G4 topology changes. The molar ratio oligonucleotide:compound was 1:4.

We next investigated *Mtb* G4 sequences in the presence of G4 ligands that have been reported to specifically recognize and stabilize G4 structures over double- and single-stranded nucleic acids. In particular, we tested a commercially available G4 ligand, BRACO-19^65^, and a newly synthesized compound, c-exNDI 2^21^, both of which have shown high selectivity for tetraplex structures over duplex. The effect of the two G4 ligands on the selected sequences in the presence of 100 mM K^+^ was initially assessed by CD analysis: they induced mild conformational changes in *Mtb* G4s without affecting the main topology, which remained characteristic of the G4 conformation (Fig. 2).

G4 ligand-induced stabilization was assessed by CD thermal unfolding analysis. G4 ligands were able to highly stabilize *Mtb* G4s with T_m_ values in some cases higher than 90 °C (Table 3). In cases where several transitions were observed (Supplementary Figures 2 and 3), T_m_ values for each transition were reported (Table 3). *zwf1* G4 was the most efficiently stabilized sequence with an increase of T_m_ higher than 41.5 °C in the presence of both BRACO-19 and c-exNDI 2 (Table 3).

**Figure 3:**
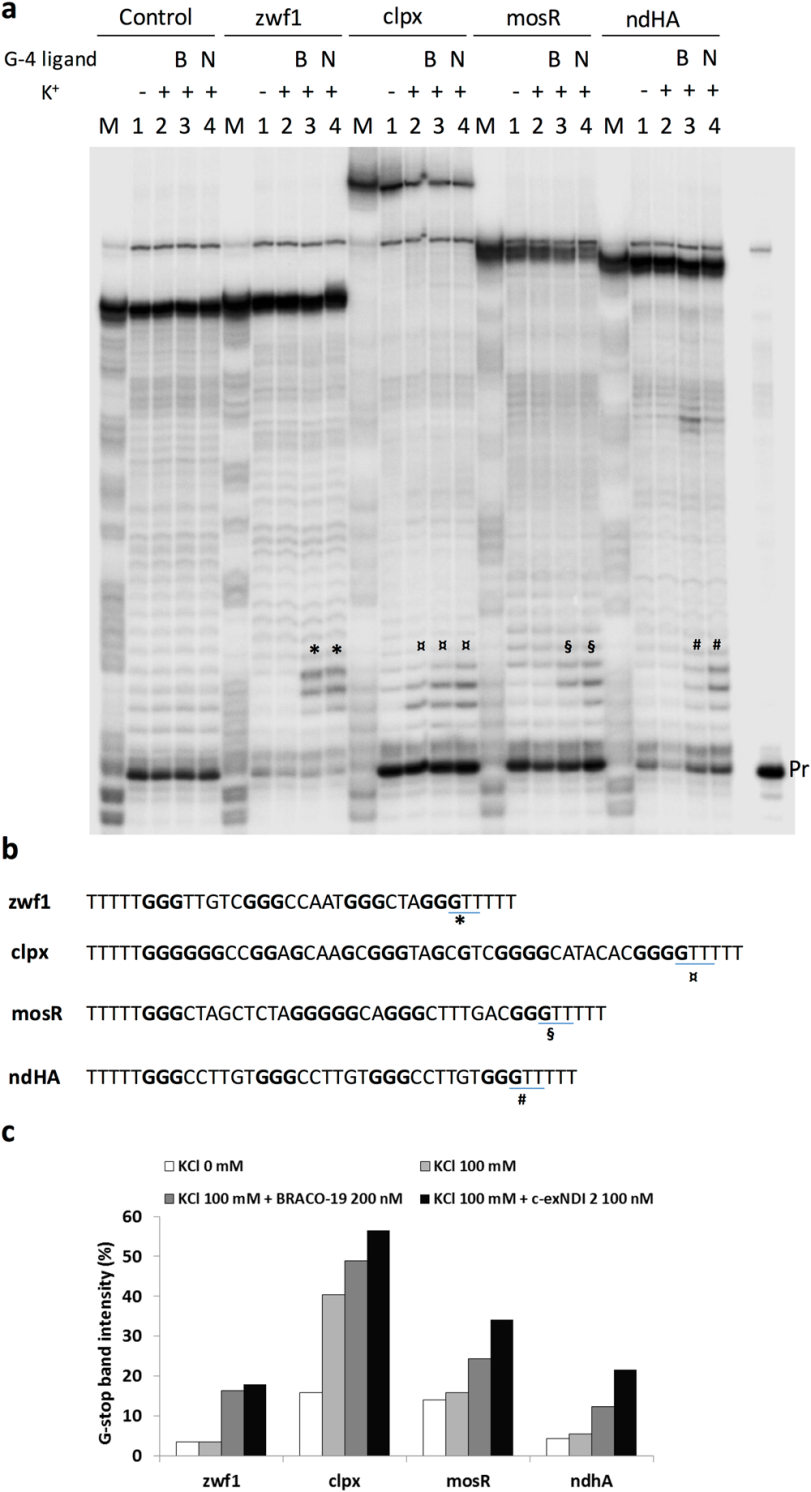
*Taq* polymerase stop assay. **(a)** Sequencing PAGE of *Taq*-amplified *zwf1*, *clpX*, *ndhA* and *mosR* templates in the absence (lanes 1) or presence of 100 mM KCl (lanes 2) and G4 ligands BRACO-19 (lanes 3) or c-exNDI 2 (lanes 4). The control template is a sequence unable to fold in G4. Symbols *, ¤, § and # indicate pausing sites just before the G4 region of the templates. Pr indicates the band of the labeled primer. M is a marker lane obtained with the Maxam and Gilbert sequencing protocol. B and N indicate BRACO-19 and c-exNDI 2, respectively. **(b)** Sequences of the selected G4 oligonucleotides. The exact position of the pausing sites within the template G4 sequence is indicated by the symbols *, ¤, § and #, as shown also in (**a**). **(c)** Quantification of the intensity of the stop bands obtained in (**a**).

G4 folding of *zwf1*, *clpX*, *mosR* and *ndhA* sequences in the absence/presence of G4 ligands was additionally tested by the *Taq* polymerase stop assay (Fig. 3). This technique allowes to evaluate G4 formation in a DNA template and G4 involvement in arresting the *Taq* polymerase processing. This G4-specific block can be then accurately solved in a denaturing polyacrylamide gel in terms of intensity and position in the sequence.

For this purpose, the *zwf1*, *clpX*, *mosR* and *ndhA* oligonucleotides were added of a primer anneali﻿ng region at their 3′-end. Moreover, additional T-flanking bases at both 5′- and 3′-ends were added to separate the 3′-end of the primer and the first G of the G4 portion. Samples were incubated in the absence or presence of 100 mM KCl (Fig. 3a, lanes 1 and 2, respectively), and with 200 nM BRACO-19 or 100 nM c-exNDI 2 (Fig. 3a, lanes 3 and 4, respectively). A control template unable to fold into G4 was also used to exclude unspecific inhibition of the polymerase enzyme by the G4 ligands. *Taq* polymerase was tested at 47 °C on all DNA templates. In the presence of all *Mtb* G4 templates, G4 ligands blocked enzyme processing (Fig. 3a,*, ¤, § and # symbols in lanes 3–4). Stop sites resulted specific and located at or just before the first 5′ G-tract involved in G4 folding (Fig. 3b). No stop site was detected on the negative control template (Fig. 3a). Quantitative analysis of G4 stop bands showed increased G4 formation in the presence of G4 ligands for all G4-forming sequences (Fig. 3c). Taken together these data indicate that the tested G4 binders strongly recognize and stabilize *Mtb* G4 sequences.

### Effect of G4 ligands on *Mtb* growth

**Figure 4:**
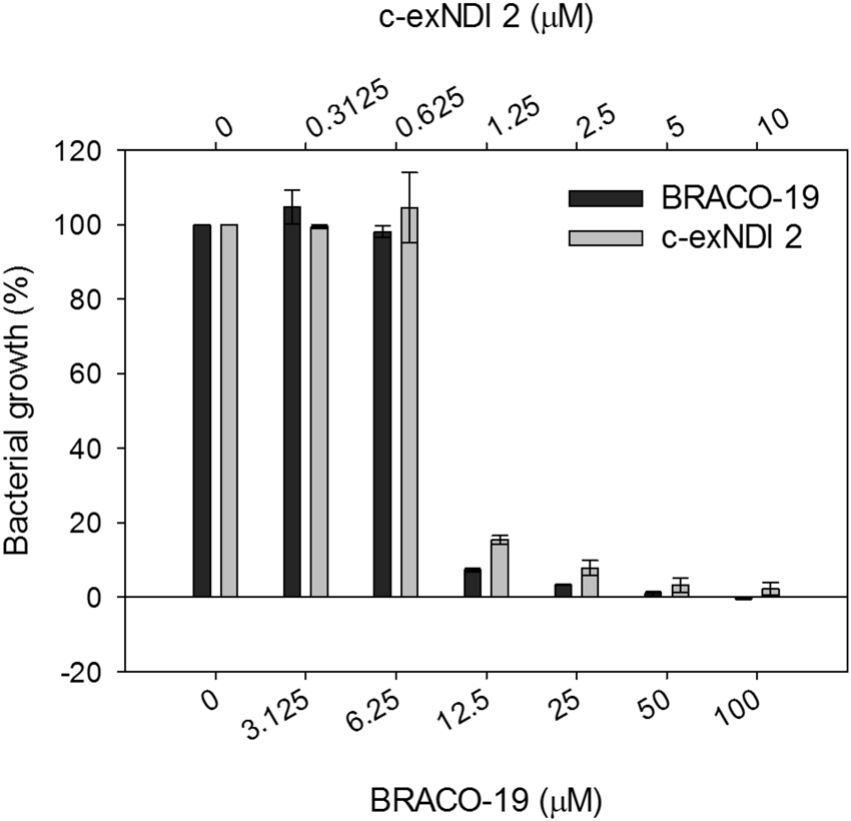
Resazurine Microplate Assay to measure the activity of different G4 ligands (BRACO- 19 and c-exNDI2) on *Mtb*.

The effect of BRACO-19 and c-exNDI 2 on *Mtb* growth was analyzed using a REsazurine Microplate Assay (REMA). As shown in Fig. 4, both compounds were able to inhibit bacterial cell growth with minimal inhibitory concentrations (MIC_80_) in the micromolar range; c-exNDI 2 was 10 times more potent than BRACO-19 with an MIC_80_ of 1.25 μM *vs* 12.5 μM. The increased potency of c-exNDI 2 may be at least in part due to its higher efficiency in stabilizing *Mtb* G4s (Table 3). However, the intracellular concentration reached by these compounds under the investigated conditions is not known. Interestingly, at least for BRACO-19, the MIC_80_ was lower than the toxic concentration for eukaryotic cells^20^ supporting the possibility to use G4 ligands to develop new antitubercular agents.

## Conclusions

Among the identified putative G4s in the *Mtb* genome, we selected 45 of them which were localized upstream of confirmed TSS and formed by at least 3 Gs in each island. The genes with predicted G4s in their TSS were distributed in several functional gene categories. Four putative G4s were selected for further characterization: we showed that all of them actually folded and were stabilized by two G4 ligands. Interestingly, the two ligands were able to inhibit *Mtb* growth *in vivo*. Our data support the possibility of *Mtb* G4 formation *in vivo* and their role as potential modulators of gene expression. Finally, our data suggest the possibility to use G4s as novel targets to develop antitubercular agents with a new mechanism of action.

## Materials and Methods

### Bioinformatics prediction of putative G4 motifs in the *Mtb* genome

An algorithm for the detection of putative G4 motifs was developed in house using Perl programing language and was applied to the reference genome of *Mtb* H37Rv (NC_000962.3). First, all guanine homopolymers (G-islands) were identified through pattern matching with the following line of code: (equation I)

$${\textstyle \text{I)}}\,seq=\sim /(G\{l,\}/g$$

where *seq* is the complete genome of *Mtb* and *l* is the minimum length required for the homopolymer. A putative G4 was reported when at least four G-islands were detected and the distance between consecutive homopolymers (loop region) was less than or equal to an additional parameter *d* (distance). G4s in the reverse strand were searched considering cytosines (C) in the same reference sequence.

In order to rank the identified G4s and focus only on those with the highest folding probability, we implemented a score measure as reported by Beaudoin *et al*.^61^. This score evaluates the presence and the relative positioning of cytosines (C) in the flanking regions surrounding a G4 motif and within the loops, since runs of consecutive ‘Cs’ were demonstrated to impair the folding of G4 structures by sequestering the ‘Gs’ in canonical Watson-Crick pairing. The score was calculated as follows (equation II):

$${\textstyle \text{II)}}\,G4\,score=\frac{cG\,score}{cC\,score}$$

cG and cC scores are defined as (equations III and IV):

$$\begin{array}{c}\mathrm{III})\quad cG(s)=\sum _{i=1}^{n}(|Gs(i)|\,\ast \,10\,\ast \,i)\\ \mathrm{IV})\quad cC(s)=\sum _{i=1}^{n}(|Cs(i)|\,\ast \,10\,\ast \,i)\end{array}$$

where ‘Gs(i)’ is the set of substrings of consecutive ‘Gs’ found in the string *s*, and |Gs(i)| is the cardinality of the set. A short and a long score were calculated, considering the G4 regions 15 or 50 nucleotides upstream and downstream.

The genomic coordinates of the predicted G4s were then intersected with promoter regions. To this aim, the list of primary TSS^62^ was exploited to extract putative promoters, which were considered embedded in the 50 nts upstream of each TSS (downstream for TSS in the reverse strand). A G4 was deemed associated to a TSS when at least one nucleotide of the G4 overlapped with the promoter. A list of all potential G4s associated to promoters is provided in Supplementary File S1.

### Oligonucleotides

All oligonucleotides used in this study were from Sigma-Aldrich (Milan, Italy) (Supplementary Table S1). BRACO-19 was from ENDOTHERM, (Saarbruecken, Germany), c-exNDI-2 was synthetized by Dr﻿. Filippo Doria and﻿ Prof. Mauro Freccero (University of Pavia).

### CD spectroscopic analysis

For CD analysis, all DNA oligonucleotides were diluted to a final concentration of 4 μM in lithium cacodylate buffer (10 mM, pH 7.4) and, where appropriate, KCl (50–150 mM). After annealing (95 °C for 5 min), all samples were gradually cooled to room temperature and compounds added from stock at final concentration of 16 µM. CD spectra were recorded on a ChirascanTM-Plus (Applied Photophysisics, Leatherhead, UK) equipped with a Peltier temperature controller using a quartz cell of 5-mm optical path length and an instrument scanning speed of 50 nm/min over a wavelength range of 230–320 nm. The reported spectrum of each sample, representing the average of 2 scans, is baseline-corrected for signal contributions due to the buffer. Observed ellipticities were converted to mean residue ellipticity (θ) = deg × cm2 × dmol−1 (mol. ellip.). For the determination of T_m_, spectra were recorded over a temperature range of 20–90 °C, with temperature increase of 5 °C/min. T_m_ values were calculated according to the van’t Hoff equation, applied for a two state transition from a folded to unfolded state, assuming that the heat capacity of the folded and unfolded states are equal.

### *Taq* polymerase stop assay

*Taq* polymerase stop assay was carried out as previously described^10^. Briefly, the 5′-end labelled primer was annealed to its template (Supplementary Table S1) in lithium cacodylate buffer in the presence or absence of KCl 100 mM and by heating at 95 °C for 5 min and gradually cooling to room temperature. Where specified, samples were incubated with BRACO-19 (250 nM) or c-exNDI-2 (100 nM). Primer extension was conducted with 2 U of AmpliTaq Gold DNA polymerase (Applied Biosystem, Carlsbad, California, USA) at 47 °C for 30 min. Reactions were stopped by ethanol precipitation; primer extension products were separated on a 16% denaturing gel, and finally visualized by phosphorimaging (Typhoon FLA 9000).

### *Mtb* strains and growth conditions

*Mtb* strain H37Rv was grown at 37 °C in Middlebrook 7H9 containing 0.5% glycerol and supplemented with 10% bovine serum albumin (BSA) – D-dextrose – NaCl (ADN), 0.05% Tween 80. Middlebrook 7H10 medium supplemented with ADN and glycerol was used as solid medium.

### REsazurine Microtiter Assay (REMA)

Drug sensitivity was determined using REMA as previously described^66^. Briefly, frozen stock cultures were grown on solid medium 7H10/ADN. Subsequently, a pre-culture was carried out in 2 ml of liquid medium (7H9/ADN) starting from an OD_540_ of 0.05. Cultures were then grown up to mid-exponential phase (OD_540_ 0.6–0.8) and then diluted to an OD_540_ of 0.01. Microplates suitable for fluorescence reading (96-well FluoroNunc^TM^ black flat bottom plates) were used to determine the MIC of each bacterial strain. Serial dilutions were used to dispense the correct amount of each compound in each well. Each well was than inoculated with a bacterial suspension containing 5 × 10^4^ cfu. The plates thus obtained were sealed and incubated for 1 week at 37 °C. After incubation, 10 µl (10% of final volume) of Alamar-Blue (Invitrogen) was added to each well and the plates, after another day of incubation at 37 °C, were read on a microplate reader (Tecan Infinite 200 Pro) to determine the relative fluorescence (excitation 535 nm and emission 590 nm). For each strain we used a positive control (cells without antibiotic) to determine the maximum fluorescence that could be obtained, and a negative control (medium plus antibiotic without cells).

## Electronic supplementary material

Supplementary Materials

Supplementary File S1a

Supplementary File S1b

Supplementary File S2
