# Supplementary material for: Mapping and characterization of G-quadruplexes in Mycobacterium tuberculosis gene promoter regions
Source: Sci Rep. 2017 Jul 18;7:5743. doi: 10.1038/s41598-017-05867-z (PMC5515968; doi:10.1038/s41598-017-05867-z)
Supplement: Supplementary file 1 — Supplementary Materials [file 41598_2017_5867_MOESM1_ESM.pdf]

Mapping and characterization of G-quadruplexes in *Mycobacterium tuberculosis* gene promoter regions

Rosalba Perrone,[§1] Enrico Lavezzo,[§1] Erika Riello[1], Riccardo Manganelli[1], Giorgio Palù[1], Stefano Toppo[1]*, Roberta Provvedi,[2]* Sara N. Richter[1]*

[1]Department of Molecular Medicine, University of Padua, via Gabelli 63, 35121 Padua, Italy

[2]Department of Biology, University of Padua, via Ugo Bassi 58/b, 35121 Padua, Italy

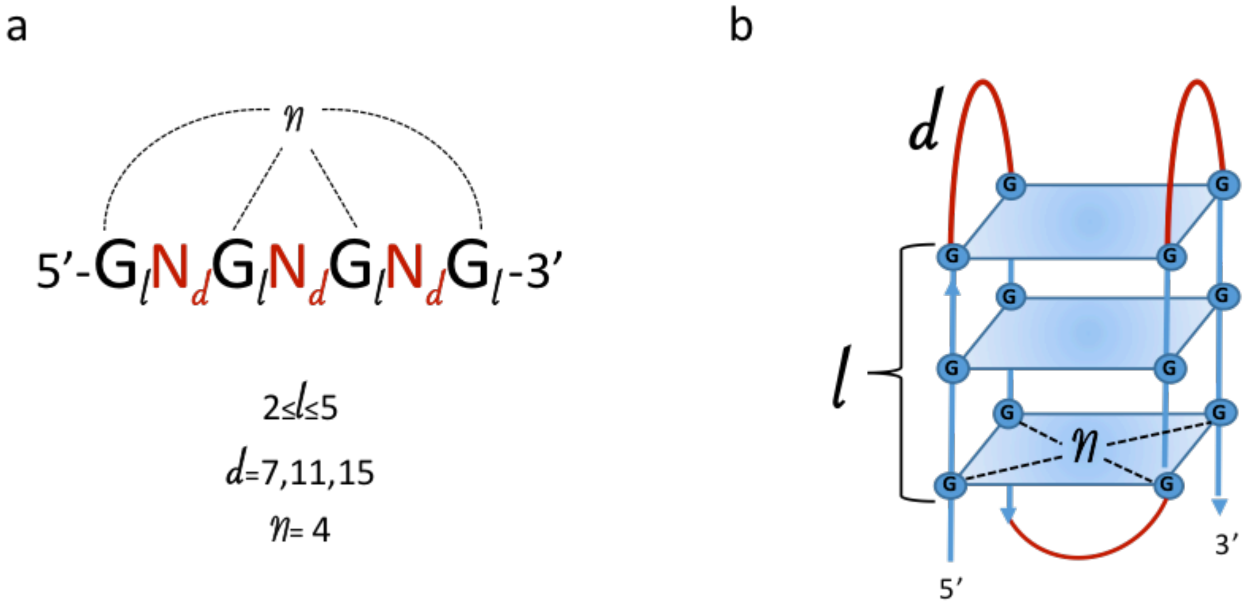

Supplementary Figure S1: Definition of parameters considered for G4 detection. n = number of G-tracts necessary to form a G4; l = length of each G-tract; d = maximum distance between two consecutive G-tracts. The parameters are shown in both the linear nucleotide sequence (a) and the three-dimensional G4 structure (b). G indicates guanine base, N indicates any nucleotide.

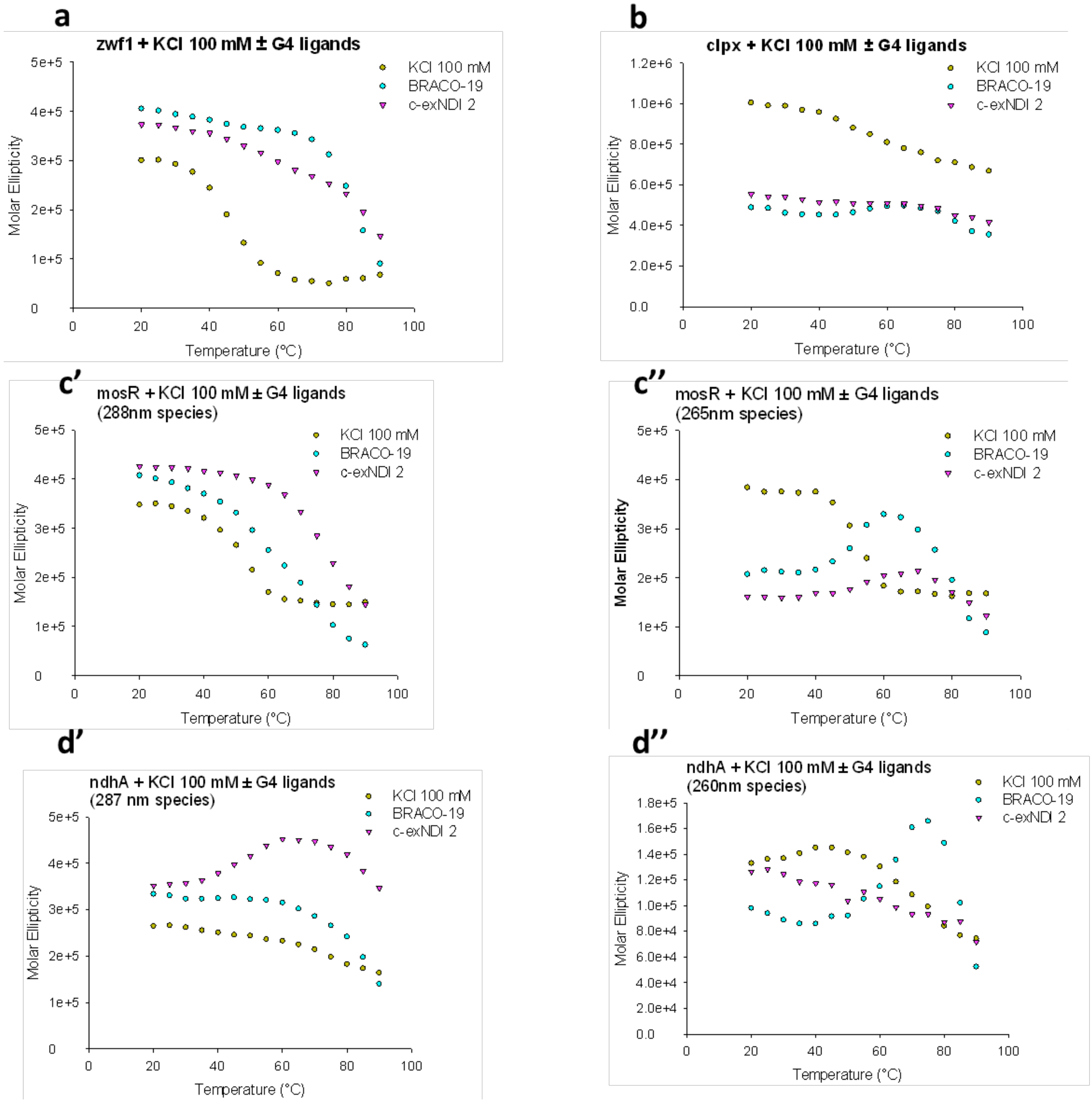

Supplementary Figure S2: CD melting curves of oligonucleotides containing G4s of *zwf1* (a), *clpx* (b), *mosR* (c’-c’’) and *ndhA* (d’-d’’) in the presence of 100 mM KCl and G4 ligands (BRACO-19 and c-exNDI 2) to assess G4 thermal stability.

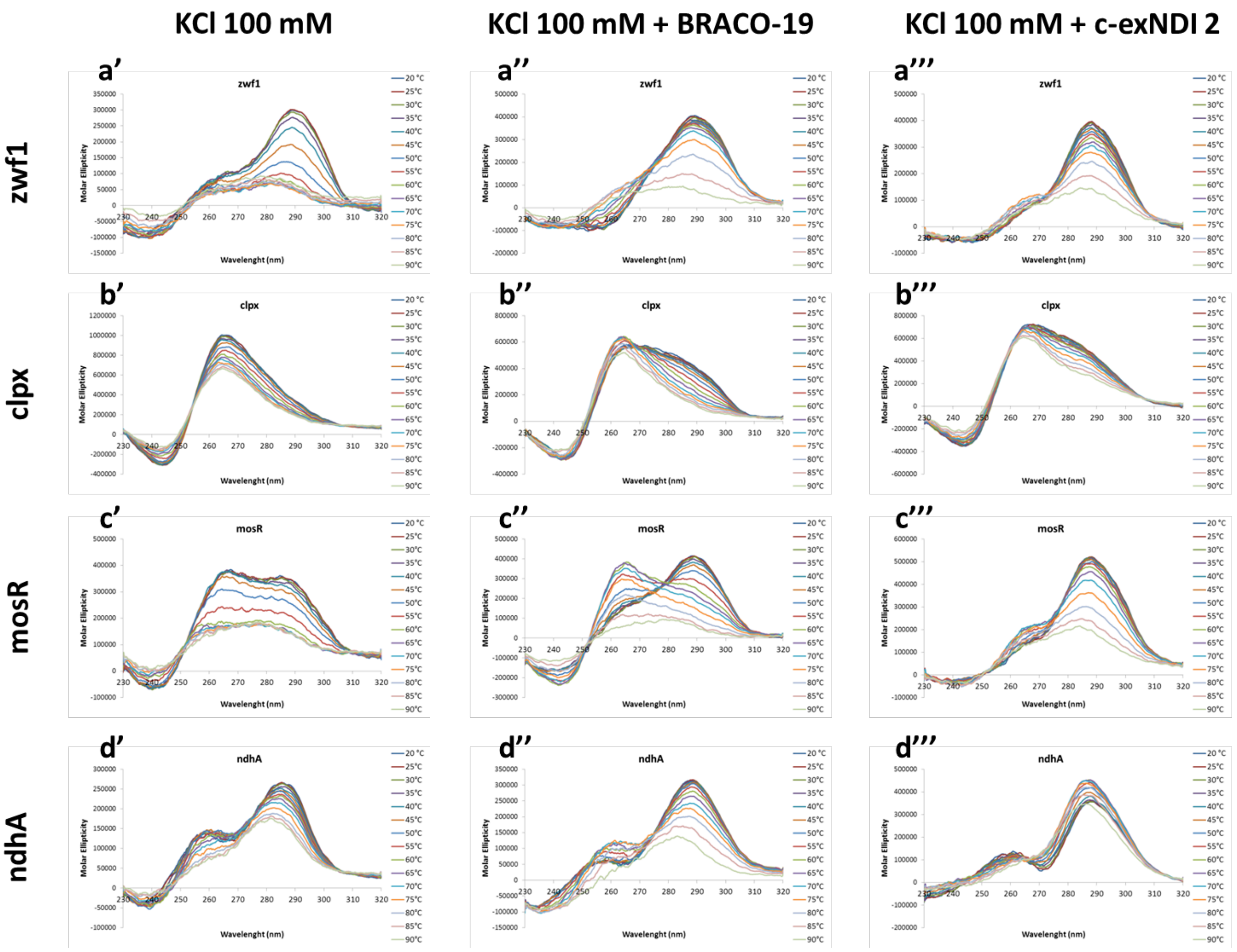

Supplementary Figure S3: CD melting spectra of *Mtb* G4 oligonucleotides in the presence of 100 mM KCl (a’-d’), BRACO-19 (a’’-d’’) or c-exNDI 2 (a’’’-d’’’) to assess G4 thermal stability and possible changes in G4 topology.

Table S1. Oligonucleotides used in CD spectroscopic analysis and *Taq* polymerase stop assay

**Applications**	**Name**	**Sequence 5’-3’**
CD assay	zwf1	TGGGTTGTCGGGCCAATGGGCTAGGGT
	clpx	TGGGGGGCCGGAGCAAGCGGGTAGCGTCGGGGCATACACGGGGT
	mosR	TGGGCTAGCTCTAGGGGGCAGGGCTTTGACGGGT
	ndhA	TGGGCCTTGTGGGCCTTGTGGGCCTTGTGGGT
Taq polymerase stop assay	Taq Primer	GGCAAAAAGCAGCTGCTTATATGCAG
	No G4	TTGTCGTTAAAGTCTGACTGCGAGCTCTCAGATCCTGCATATAAGCAGCTGCTTTTTGCC
	zwf1	TTTTTGGGTTGTCGGGCCAATGGGCTAGGGTTTTTCTGCATATAAGCAGCTGCTTTTTGCC
	clpx	TTTTTGGGGGGCCGGAGCAAGCGGGTAGCGTCGGGGCATACACGGGGTTTTTCTGCATATAAGCAGCTGCTTTTTGCC
	mosR	TTTTTGGGCTAGCTCTAGGGGGCAGGGCTTTGACGGGTTTTTCTGCATATAAGCAGCTGCTTTTTGCC
	ndhA	TTTTTGGGCCTTGTGGGCCTTGTGGGCCTTGTGGGTTTTTCTGCATATAAGCAGCTGCTTTTTGCC
